# Analysis of Gender Dimorphism and Assessment of Racial Variation Through Odontometric Technique: A Cross-Sectional Study

**DOI:** 10.7759/cureus.51446

**Published:** 2024-01-01

**Authors:** Namdeo Prabhu, Rakhi Issrani, Krishna Rao, Ahmed Saleh Albalawi, Bader Mahali Alharbi, Abdulelah Waheed Noman Alanazi, Mohammad Khursheed Alam

**Affiliations:** 1 Oral and Maxillofacial Surgery and Diagnostic Sciences, College of Dentistry, Jouf University, Sakaka, SAU; 2 Preventive/Oral Medicine & Radiology, College of Dentistry, Jouf University, Sakaka, SAU; 3 Preventive Dental Science, College of Dentistry, Jouf University, Sakaka, SAU; 4 Dentistry, College of Dentistry, Jouf University, Sakaka, SAU; 5 Orthodontics and Dentofacial Orthopedics, College of Dentistry, Jouf University, Sakaka, SAU

**Keywords:** dentistry, tooth crown, odontometric, ethnic groups, dentition

## Abstract

Background: When conducting a forensic investigation, one of the most important steps is establishing the biological profile of a victim who cannot be positively recognized or is just a skeleton. It has been shown that, among the other clinical indicators, the diameters of dental crowns are a good and dependable source for determining gender in a particular population sample. However, the literature is sparse regarding their assessment as a viable marker for the determination of a particular race. In addition, the need for population-specific data has also been advocated while determining gender dimorphism based on tooth size.

Aim and objectives: To study the bisexual variation in the permanent dentition of individuals from three different sets of populations: Arabian, South Asian, and East Asian. The other objective is to explore the role of this odontometric analysis in predicting the racial identity of the subjects belonging to the aforementioned population.

Methodology: The research was conducted at the College of Dentistry, AlJouf University in Sakaka. Measurements of mesiodistal and buccolingual (BL) distances were taken using a digital vernier caliper on a total of 75 pairs of research models or casts. Statistical tests were run on the information gathered.

Results: Of the 75 casts, 38 (50.7%) were of male and 37 (49.3%) were of female. Our analysis showed between genders, a significant difference in maxillary central incisor (P = 0.001), first premolar (P = 0.01), and first molar (P = 0.02) while for a mandibular arch, a significant difference was noted for incisors (P = 0.002) with greater tooth dimension in male than in the female. Concerning the BL dimensions, only the mandibular canine showed a significant difference between males and females (P = 0.001). Comparisons of the crown dimensions between population groups showed that the Arabian population consistently exhibits larger tooth dimensions than the other two populations in both arches.

Conclusion: A few crown dimensions can be used as an adjunctive tool for the identification of the gender and race of an individual.

## Introduction

Recent advances in the science of understanding the function of teeth have created enormous relevance in the identification of race and gender across animals and communities. Due to the damaged condition of the soft tissues, identification of human remains following mass catastrophes is often limited to bones and teeth [1]. The field of forensic dentistry has been crucial in the identification of victims of major tragedies [2]. Because of its unique physical properties, the dentition is frequently able to survive catastrophic events while the underlying bone components of the body are destroyed [3]. Because of its unique physical properties, dentition is frequently able to survive catastrophic events while the underlying bone components of the body are destroyed [4]. The use of dental morphology to detect sexual dimorphism has played a crucial role in postmortem destruction and fragmentation [5].

Anthropologists and forensic dentists have long been curious about the phenomenon of sexual dimorphism in tooth size [1-4]. However, the literature is sparse regarding their assessment as a viable marker for the determination of a particular race. Studies show that teeth are an excellent adjunct for sex differences, even though they cannot be utilized as the primary predictor of gender [1]. It has been argued that the greatest number of anatomical factors should be used for determining the gender of the skeleton [6]. Forensic sex identification aided by teeth is thus useful, especially in cases when other, more trustworthy diagnostic characteristics have been lost or deteriorated [1].

In the last two decades, odontometrics has been investigated as a method for determining racial and gender identity in the forensics literature [2-4,7-9]. Examiners care most about the linear measures of the mesiodistal (MD) and buccolingual (BL) dimensions of the teeth. While it would be preferable to measure both, it is not unusual to encounter instances where just one has been tested [2,7,10]. Population variance is most noticeable when comparing very different ethnic groups (such as Europeans and Asians), although there is still considerable room for error even among more closely related groups. A person's jaw and side might also affect their dependability [11]. Potter [12] argues that the left side is more correct than the right, even though that orientation is often disregarded [1,2,10]. There is more sexual dimorphism in the mandibular teeth, according to several studies examining the jaw [12-14], but different populations exhibit the opposite [2,10].

The purpose of this study was to examine the relationship between odontometric measurements and gender classification and to determine the accuracy with which these measures could be employed for the determination of race between three different sets of populations, namely the Arabian, South Asian, and East Asian.

## Materials and methods

Study design

This research examines the correlation between odontometric variables and self-reported gender in three distinct populations through a cross-sectional design (April 2023 to June 2023). All research techniques complied with the 2013 edition of the Helsinki Declaration and received clearance from Jouf University's local bioethics committee (approval no. 12-16-8/39). After thoroughly outlining the reasons for and desired outcomes of the therapy, informed permission was gained from the patient at that time.

Sample population, size, and characteristics

The study sample comprised 75 pairs of study models or casts. Some of them were collected in the past from patients who were seen in the outpatient department, while others were from people who are now receiving care at the College of Dentistry, AlJouf University, Sakaka. Three things were taken into account: 1) the lack of any obvious dental abnormalities; 2) the lack of any conservative treatment other than Class I preparation; and 3) the presence and full eruption of all morphologically normal permanent teeth except for the third molars in both arches. The exclusion criteria were 1) patients who were undergoing orthodontic and prosthodontic treatment; 2) patients allergic to alginate impression material; and 3) models displaying casting defects affecting the crowns of the teeth.

Both sexes were represented in this sample of 500 people from the ages of 17 to 35 from the Arabian (including native Saudi Arabians and Egyptians), South Asian (including Indians, Pakistanis, and Bangladeshis), and East Asian (including Filipinos) regions. Due to the fact that teeth do not display remodeling as in bone and thus stay unaltered other than attrition and other dental illnesses, individuals within this age range were considered eligible for cross-sectional research of this kind.

The interviewers did not utilize a questionnaire to determine the participants' ancestry and ethnicity. To prevent bias during the selection process, each participant's name, Date of birth (DOB), gender, and race were randomly assigned numbers on the computer, and the research models were given the same numbers.

Studied parameters

MD Diameter of Crown

According to Hunter et al., this is the longest distance (in millimeters) between the contact sites of teeth on each side of the jaw when the caliper beaks are held perpendicular to the occlusal and buccal surfaces [15].

BL Diameter of the Crown

**Figure 1:**
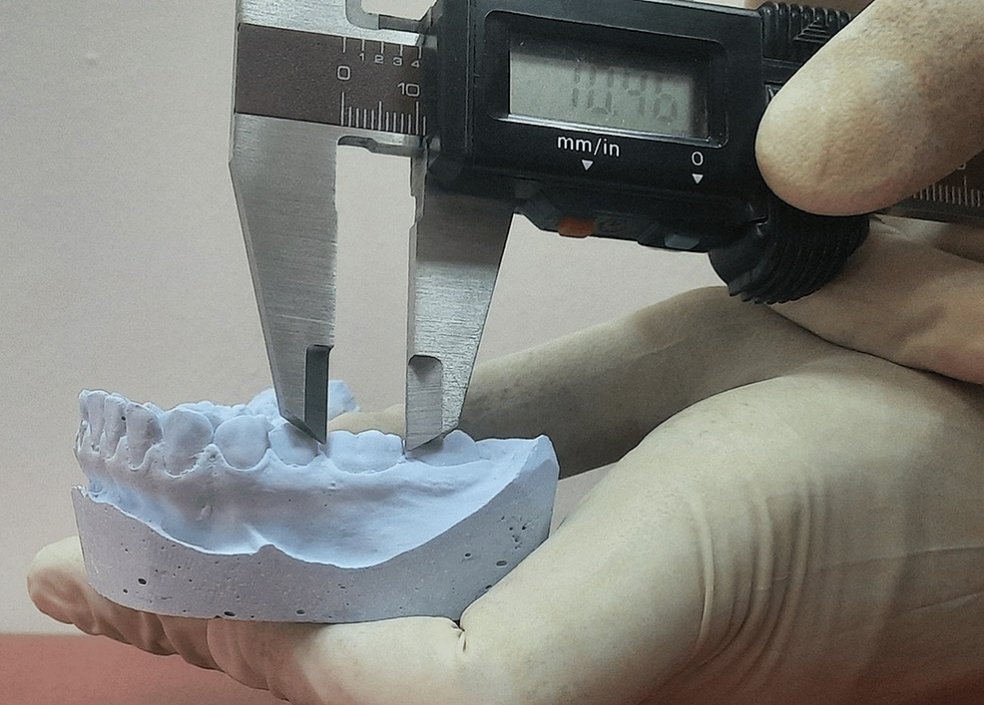
Measurement of mesio distal diameter of crown

**Figure 2:**
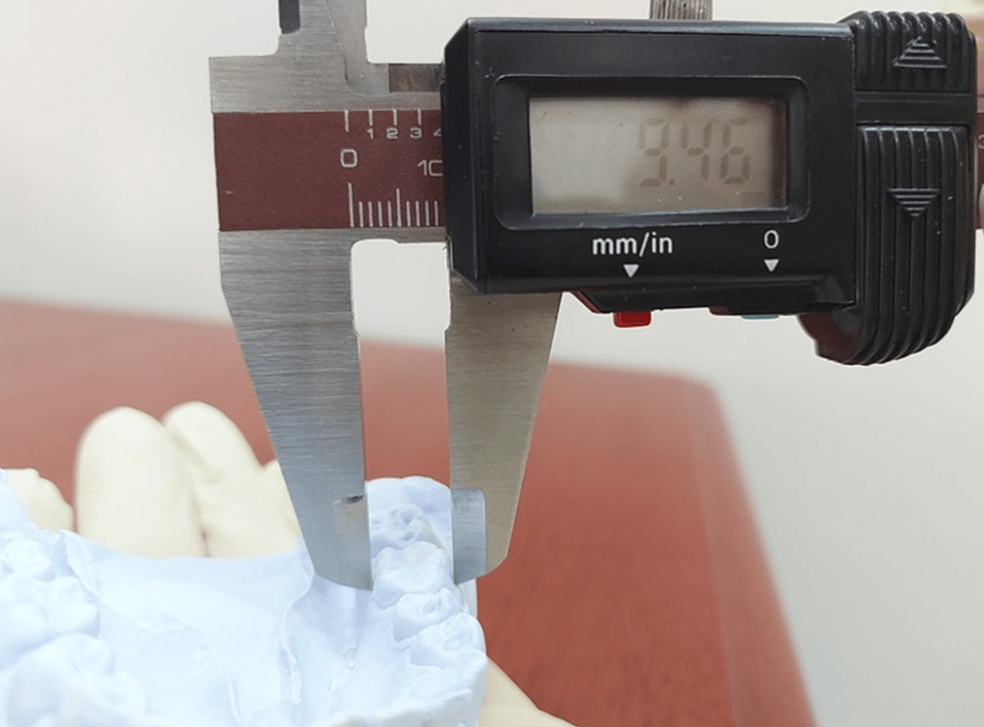
Measurement of BL diameter of crown

According to Seipel Seipel Cognitive Model (CM's) definition, this is the distance between the buccal and lingual surfaces of the crown when measured perpendicular to the plane in which the MD diameter is determined [16] (Figure 1, 2).

Procedure

Patients' informed consents were obtained before the clinical assessment, and then alginate imprints of the maxillary and mandibular arches were produced in perforated trays (Nos. 2, 3, and 4). The study was approved by the institutional ethical clearance committee. To prevent excessive shrinkage, dental stones of type IV were put into alginate molds as soon as they were taken. An excess dental stone was cut out of the dental cast. After at least 24 hours, the MD and BL dimensions of the left quadrant of the maxillary and mandibular dentitions were measured directly on the castings with a digital vernier caliper (resolution 0.01 mm). It was made sure that the teeth used to take the measurements were generally undamaged and healthy, to provide “unaltered” anatomic data. Measurements were obtained between places on the proximal surfaces of the crown where it was assumed contact with neighboring teeth would have occurred when teeth were rotated or misaligned. While this criterion was strictly adhered to, the actual measurements taken only went as far as the caliper could reach, which may not be the same as the anatomical crest of curvature on the tooth surfaces. There were, however, a few cases of restorations, cavities, partial dentures, severe wear, or casting faults among the castings. Bilateral impediments that made it difficult to take dental measurements were excluded from the study. However, in a few instances, the contralateral teeth belonging to the same arch were utilized for the analysis when they were free of such defects and also in cases where teeth were missing or extracted in the quadrant that was subjected to the analysis.

The measurements were performed independently by the investigators, who were randomly categorized into two groups, and all values were taken to two decimal places. As and when the measurements were completed, the study models were exchanged between the two groups and measured again to ensure the reliability of the measurements. The levels of agreement between the two sets of testers were 0.95 for intra-examiner testing and 0.96 for inter-examiner testing, as calculated by Cohen's Kappa.

Statistical analysis

The data were analyzed using SPSS 21.0 (IBM Corp., Armonk, NY, USA). Descriptive statistical methods (mean and standard deviation) were used for the evaluation of the data. Comparative analysis for crown dimensions between permanent dentition and population groups was tested using ANOVA, whereas crown dimensions between genders were done using the student t-test. A P-value of ≤ 0.05 was set as a statistically significant value.

## Results

Tooth surface comparisons

The data on MD and BL crown dimensions in millimeters (mm) of the permanent maxillary and mandibular teeth studied is summarized in Table 1. A statistically significant difference was noted between the two arches (P = 0.001).

**Table 1:** Comparative analysis for crown dimensions of permanent dentition of study groups I1 = Central incisor; I2 = Lateral incisor; C = Canine; PM1 = First Premolar; PM2 = Second Premolar; M1 = First Molar; M2 = Second Molar; BL = Buccolingual; MD = Mesiodistal

Arch	Tooth	Surface	Mean (mm)	Standard deviation	Minimum	Maximum	ANOVA	P-value
M A X I L L A R Y	I1	BL	6.8	.78	5.0	8.6	208.82	0.001
		MD	8.2	0.62	6.7	9.8		
	I2	BL	6.1	0.84	4.4	8.1		
		MD	6.2	0.62	4.2	7.6		
	C	BL	7.5	0.88	6.0	9.4		
		MD	7.2	0.79	3.8	8.5		
	PM1	BL	8.8	0.82	7.4	11.1		
		MD	6.7	0.61	4.8	8.5		
	PM2	BL	9.1	0.98	7.0	12.2		
		MD	6.4	1.05	4.5	10.8		
	M1	BL	10.9	0.81	8.1	12.4		
		MD	9.6	1.19	5.1	12.0		
	M2	BL	10.8	0.89	8.5	12.8		
		MD	9.4	0.65	7.8	10.4		
M A N D I B U L A R	I1	BL	5.6	0.62	3.9	7.6		
		MD	5.2	0.49	4.1	6.5		
	I2	BL	5.7	0.61	3.8	7.0		
		MD	5.5	0.66	3.8	7.0		
	C	BL	6.8	0.83	5.2	9.0		
		MD	6.3	0.60	4.7	8.0		
	PM1	BL	7.6	0.65	5.2	9.2		
		MD	6.5	0.64	4.6	8.0		
	PM2	BL	8.6	3.96	6.2	42.0		
		MD	6.6	0.65	4.8	7.9		
	M1	BL	10.4	0.81	7.1	12.2		
		MD	10.1	1.08	5.3	11.9		
	M2	BL	10.2	0.84	8.0	12.3		
		MD	9.9	0.66	8.9	12.0		

Male/female comparisons

Of the 75 cast members, 38 (50.7%) were male and 37 (49.3%) were female. The mean (±SD) age of the male patients was 29.3 (±6.2) years, whereas in females it was 28.9 (±6.5) years. Males exhibited larger tooth dimensions than females.

MD dimensions

Our analysis showed a significant difference between genders in the maxillary central incisor (P = 0.001), first premolar (P = 0.01), and first molar (P = 0.02) while for the mandibular arch, a significant difference was noted for the incisors (P = 0.002) with greater tooth dimension in males than females. The largest sexual dimorphism in the MD crown dimension was exhibited by the mandibular lateral incisor (0.4 mm). The mean MD crown dimension of the maxillary canines was greater than that of the mandibular canines, with an average of 1.0 mm in males and 0.9 mm in females. In both sexes, the mean MD crown dimension of the first premolars was larger than that of the second premolars for both arches.

BL Dimensions

Concerning the BL dimensions, only the mandibular canine showed a significant difference between males and females (P = 0.001) (Table 2).

**Table 2:** Descriptive statistics for crown dimensions between genders I1 = Central incisor; I2 = Lateral incisor; C = Canine; PM1 = First Premolar; PM2 = Second Premolar; M1 = First Molar; M2 = Second Molar; BL = Buccolingual; MD = Mesiodistal; SD = Standard Deviation; N = Number of participants

Arch	Tooth	Surface	Male (N=38)	Female (N=37)	Student t	P-value
			Mean±SD	Mean±SD		
MAXILLARY	I1	BL	6.8±.80	6.8±0.76	0.14	0.71
		MD	8.2±.77	8.1±0.44	16.8	0.001
	I2	BL	6.1±.84	6.0±0.84	0.26	0.60
		MD	6.4±.55	6.1±0.67	0.28	0.59
	C	BL	7.5±.95	7.4±0.83	0.98	0.32
		MD	7.3±.63	7.2±0.93	2.4	0.12
	PM1	BL	9±.84	8.7±0.76	0.35	0.55
		MD	6.7±.40	6.7±0.77	12.1	0.01
	PM2	BL	9.1±1.03	9.1±0.93	0.82	0.37
		MD	6.5±.99	6.2±1.09	0.08	0.76
	M1	BL	10.9±.82	10.7±0.79	0.00	0.97
		MD	9.7±1.37	9.5±0.99	4.9	0.02
	M2	BL	10.9±.89	10.6±0.87	0.04	0.84
		MD	9.5±.68	9.3±0.63	0.36	0.85
MANDIBULAR	I1	BL	5.7±.67	5.5±0.54	2.82	0.09
		MD	5.3±.37	5.1±0.57	10.19	0.002
	I2	BL	5.8±.50	5.6±0.70	3.17	0.79
		MD	5.7±.76	5.3±0.52	10.15	0.002
	C	BL	6.9±.99	6.6±0.62	15.55	0.001
		MD	6.3±.50	6.3±0.69	3.34	0.72
	PM1	BL	7.6±.75	7.6±0.54	1.65	0.20
		MD	6.6±.53	6.5±0.63	1.02	0.31
	PM2	BL	9±.56	8.9±0.64	2.71	0.10
		MD	6.5±.58	6.5±0.73	2.38	0.12
	M1	BL	10.3±.80	10.3±0.84	1.22	0.27
		MD	10.2±1.01	10±1.14	1.44	0.23
	M2	BL	10.3±.94	10.1±0.94	1.47	0.22
		MD	9.9±.69	9.8±0.64	0.95	0.33

Geographic variations in tooth size

Comparisons of the crown dimensions between population groups showed that the Arabian population consistently exhibits larger tooth dimensions than the other two populations in both arches. The South Asian population has tooth sizes close to those of the East Asian population. Although substantial between-group tooth size differences were apparent in some instances (i.e. BL dimensions of the maxillary lateral incisor, maxillary first and second premolar; and mandibular first premolar and MD dimension of the maxillary second molar), the measurements of individual teeth were remarkably homogeneous among the various geographic regions (Table 3).

**Table 3:** Correlation coefficients between crown diameters of maxillary and mandibular teeth in different racial groups I1 = Central incisor; I2 = Lateral incisor; C = Canine; PM1 = First Premolar; PM2 = Second Premolar; M1 = First Molar; M2 = Second Molar; BL = Buccolingual; MD = Mesiodistal; SD = Standard Deviation

Arch	Tooth	Surface	Arabian		South Asian		East Asian		F	P-value
			Mean	SD	Mean	SD	Mean	SD		
M A X I L L A R Y	I1	BL	7.1	0.84	6.8	0.69	6.6	0.74	2.5	0.08
		MD	8.2	0.59	8.0	0.73	8.3	0.53	1.2	0.29
	I2	BL	6.5	0.71	5.9	1.00	5.9	0.68	3.6	0.03
		MD	6.3	0.50	6.3	0.75	6.1	0.56	1.2	0.31
	C	BL	7.6	0.82	7.4	0.94	7.5	0.91	0.34	0.70
		MD	7.3	0.94	7.2	0.78	7.2	0.64	0.15	0.86
	PM1	BL	9.2	0.66	8.8	0.58	8.5	0.99	6.2	0.001
		MD	6.7	0.60	6.6	0.71	6.7	0.52	0.01	0.98
	PM2	BL	9.5	0.84	9.1	0.85	8.7	1.11	3.9	0.02
		MD	6.5	1.13	6.2	0.90	6.5	1.10	0.97	0.38
	M1	BL	10.7	0.86	11.1	0.69	10.8	0.86	1.1	0.32
		MD	9.5	1.24	9.7	1.3	9.5	1.02	0.24	0.78
	M2	BL	11.1	0.72	10.8	0.89	10.5	1.09	2.1	0.12
		MD	9.3	0.68	9.7	0.69	9.3	0.51	3.2	0.04
M A N D I B U L A R	I1	BL	5.7	.52	5.6	0.69	5.6	0.65	0.25	0.77
		MD	5.2	0.50	5.1	0.57	5.4	0.36	1.7	0.17
	I2	BL	5.8	0.53	5.7	0.68	5.6	0.61	0.52	0.59
		MD	5.3	0.64	5.6	0.86	5.6	0.41	1.0	0.37
	C	BL	7.05	0.81	6.6	0.83	6.8	0.84	1.5	0.22
		MD	6.1	0.66	6.3	0.60	6.5	0.49	2.6	0.08
	PM1	BL	8.0	0.60	7.5	0.74	7.4	0.41	7.7	0.001
		MD	6.5	0.72	6.6	0.74	6.5	0.44	0.00	0.99
	PM2	BL	9.8	6.7	7.9	0.60	8.0	0.75	1.7	0.17
		MD	6.6	0.65	6.5	0.73	6.6	0.60	0.33	0.71
	M1	BL	10.4	0.94	10.3	0.81	10.3	0.69	0.42	0.65
		MD	9.9	1.52	9.9	0.78	10.4	0.70	1.7	0.17
	M2	BL	10.2	0.80	10.2	0.79	10.0	0.92	0.61	0.54
		MD	9.8	0.73	10.0	0.67	9.8	0.58	0.87	0.42

## Discussion

Determining an individual's gender is a crucial aspect of forensic investigations. Typically, this involves utilizing morphological characteristics and anthropometric methods. However, when dealing with fragmentary remains where anthropometric methods may have limitations due to the condition and availability of bones [2], odontometric parameters present a valuable alternative. Odontometric parameters provide a straightforward and dependable approach to gender determination. It is important to note that these parameters exhibit variations not only among different populations but also within the same population.

The process of paleo-anthropological and forensic identification of excavated human remains involves three primary steps: identification of the decedent's race, gender, and age [17]. The dentition is a useful sex predictor, especially in young people, based on the concept that most teeth grow to completion before skeletal development [18]. Because of recent years' increased focus on dentition and the fact that teeth are often the best-preserved part of the skeleton during archaeological excavations, crown dimension measurement has been employed for sex determination. In addition, measuring a patient's teeth takes very little time and does not require any drilling. Several writers have investigated ethnic differences in crown sizes, although this is not the primary focus [19]. Differences in crown diameter and shape by race would provide a sense of the relationship between populations and environmental adaptation; hence, this data are crucial.

Casts of the teeth continue to play an important role as a diagnostic tool in modern dentistry. Even though the dental cast measurements are said to be, on average, 0.1 mm bigger than those of the real teeth, examination of dental casts seems suited to this kind of inquiry since dental cast measurements are more dependable than those taken directly in the oral cavity [20].

A review of the relevant literature turns up no studies that directly compare the sizes of teeth among the three major ethnic groups. To identify anthropometric traits specific to each ethnicity that may aid in differentiation during forensic investigation, the present study analyzed and compared the MD and BL crown diameters of the three racial groups using dental casts.

Males in the current research exhibited bigger teeth across the board in terms of both upper and lower jaw dimensions, as determined by comparing the mean values of the parameters assessed across sexes. This agrees with the results found by Al-Gunaid et al. [21], Khan et al. [22], and Adeyemi et al. [23]. Some researchers believe that the Y chromosome, which boosts the mitotic capacity of the tooth germ and produces dentinogenesis, and the X chromosome, which induces amelogenesis, are to blame for the observed sex difference in tooth size [18].

The current research found evidence of sexual dimorphism in the mean dental distance (MD) of the maxillary central incisor, first premolar, and first molar, and in the mean dental distance (BL) of the mandibular incisors and canine. Consistent with the results of Shireen et al. [18], where it was discovered that MD and BL measurements of the maxillary first molars and mandibular canine may help in sex identification by Iscan et al. [2]. While this is true for the posterior teeth, it has been reported that the BL dimension may be quantified more accurately than others. Since proximal contact between teeth complicates obtaining MD measurements, they are generally considered to be less accurate than BL measurements. On the other hand, Potter RHY [12] has shown that multidimensional scaling (MD) variables are particularly helpful in stepwise discriminant analysis, with ten of the twelve variables used in his research being MD dimensions. Possible explanations for the superior sex-discriminating power of MD measurements include their association with maxillary and mandibular arch dimensions. Jaws in males are inferred to produce proportionately bigger MD dimensions on account of the greater anteroposterior jaw measures and the correlation between arch size and tooth size [18].

Furthermore, the research found that the crown dimensions of the Arabian population were consistently the largest of the three groups in both arches, whereas the South Asian and East Asian groups had similar tooth sizes. Other than for a few crown dimensions in both the arches, most of the teeth showed insignificant differences for all the populations, and hence none of the variables could be used to predict the race of an individual. This is in harmony with the findings of Adeyemi et al. [23], where there is no significant difference in tooth sizes between Nigerians and Americans. However, this is inconsistent with the findings of Ghose et al. [24]. In Iraqis and Bedouins, the scientists discovered that the breadth of people's teeth decreased significantly from northern to southern populations, with Yemenis having the smallest teeth of any of the studied groups, followed by Bedouins and Iraqis. Similarly, Al-Gunaid et al. [21] teeth size in Yemeni Arabians was found to rise gradually from south to north, with southern Yemenis having the lowest teeth, followed by middle and northern Saudis and Jordanians. Otuyemi et al. [25] compared the tooth sizes of Nigerians and Britons and found that the mean MD tooth sizes for all teeth were substantially bigger in Nigerians, although no statistically significant variations were shown between the BL crown diameters of the two groups [26]. Studies in Mexico and the US found only small differences in MD crown dimensions, and that would be of little importance in orthodontic diagnosis.

Important findings from this study include the use of tooth crown dimensions for rapid and straightforward sex determination, with measurements on dental casts proving to be more reliable than intraoral measurements. Males consistently exhibited larger teeth in all dimensions, aligning with previous research. Specifically, maxillary central incisor, first premolar, and first molar, as well as mandibular incisors' MD dimensions, and mandibular canine's BL dimensions, showed sexual dimorphism, consistent with prior studies. Notably, this study also found that Arabian population had consistently larger tooth crown dimensions compared to the other two racial groups (South Asian and East Asian), a comparison not thoroughly explored in existing literature.

It is important to note that this study's limitations, such as its small sample size, hinder the development of a reliable regression equation for predicting race based on tooth dimensions. To improve the accuracy of such analyses, future research should involve larger sample sizes and multi-center studies conducted in racially or geographically homogeneous groups. These studies should also consider potential confounding variables, including environmental factors, diet, and nutrition, which can impact tooth dimensions.

## Conclusions

The study results suggest that odontometric analysis can serve as a reliable tool for assessing sexual dimorphism in dental crown dimensions within these populations. Furthermore, the observed variations in tooth dimensions between populations, with the Arabian population consistently having larger dimensions, highlight the potential utility of dental crown measurements in predicting racial identity.

Overall, this study contributes valuable insights into the use of dental crown dimensions as a means to determine sexual dimorphism and potentially aid in racial identification within the context of forensic investigations involving skeletal remains or unidentified individuals. Further research with larger and more diverse population samples is warranted to enhance the robustness and applicability of these findings in forensic contexts.
